# The Felix-trial. Double-blind randomization of interspinous implant or bony decompression for treatment of spinal stenosis related intermittent neurogenic claudication

**DOI:** 10.1186/1471-2474-11-100

**Published:** 2010-05-27

**Authors:** Wouter A Moojen, Mark P Arts, Ronald Brand, Bart W Koes, Wilco C Peul

**Affiliations:** 1 Department of Neurosurgery, Leiden University Medical Center, Leiden, The Netherlands; 2 Department of Neurosurgery, Medical Center Haaglanden, The Hague, The Netherlands; 3 Department of Medical Statistics, Leiden University Medical Center, Leiden, The Netherlands; 4 Department of General Practice, Erasmus Medical Center, Rotterdam, The Netherlands

## Abstract

**Background:**

Decompressive laminotomy is the standard surgical procedure in the treatment of patients with canal stenosis related intermittent neurogenic claudication. New techniques, such as interspinous process implants, claim a shorter hospital stay, less post-operative pain and equal long-term functional outcome. A comparative (cost-) effectiveness study has not been performed yet. This protocol describes the design of a randomized controlled trial (RCT) on (cost-) effectiveness of the use of interspinous process implants versus conventional decompression surgery in patients with lumbar spinal stenosis.

**Methods/Design:**

Patients (age 40-85) presenting with intermittent neurogenic claudication due to lumbar spinal stenosis lasting more than 3 months refractory to conservative treatment, are included. Randomization into interspinous implant surgery versus bony decompression surgery will take place in the operating room after induction of anesthesia. The primary outcome measure is the functional assessment of the patient measured by the Zurich Claudication Questionnaire (ZCQ), at 8 weeks and 1 year after surgery. Other outcome parameters include perceived recovery, leg and back pain, incidence of re-operations, complications, quality of life, medical consumption, absenteeism and costs. The study is a randomized multi-institutional trial, in which two surgical techniques are compared in a parallel group design. Patients and research nurses are kept blinded of the allocated treatment during the follow-up period of 1 year.

**Discussion:**

Currently decompressive laminotomy is the golden standard in the surgical treatment of lumbar spinal stenosis. Whether surgery with interspinous implants is a reasonable alternative can be determined by this trial.

**Trial register:**

**Dutch Trial register number**: NTR1307

## Background

Intermittent Neurogenic Claudication (INC) is a complex of symptoms first described by Van Gelderen in 1948 and in 1950 by the Dutch neurosurgeon Verbiest, therefore formerly known as the Verbiest syndrome [1-4]. The characteristic symptom is described as leg pain (frequently in both legs) which can be exacerbated with prolonged walking or lumbar extension. Others, like Evans, describe a cramp, tightness or discomfort of the legs after walking which diminish after a short period of sitting or bending forward [1]. Apart from the leg pain, associated low back pain may occur [5].

Since the description of neurogenic claudication by Verbiest, explanation of the symptoms has been disputed. Verbiest stated in 1954: "*In the writer's humble opinion the ligamentum flavum is most unlikely to contact any spinal root unless this root is distorted from its regular path"*[4]. Evans showed in 1964 a cerebral spinal fluid stop at the low lumbar levels narrowing of the canal by degenerative facet arthrosis resulting in nerve root compression. INC is often seen in patients with lumbar degenerative spinal stenosis [4]. Due to this arthrosis of the facet joints, lumbar nerve root compression will develop. Arnoldi described multiple types of lumbar spinal stenosis. His article published in 1975 was actually a summarization of a symposium on this subject [4,6]. Presently, his classification is still widely used. Like in any acquired disease, INC is usually seen in the elderly [1].

The best treatment of NIC due to lumbal stenosis remains controversial [5,7]. Nonoperative therapy like epidural steroid injections, nonsteroidal anti-inflammatory medication, analgesics, physical therapy, and spinal manipulation, is frequently performed [8]. A 2005 Cochrane review found that the paucity and heterogeneity of evidence limited conclusions regarding surgical efficacy for spinal stenosis [7,9-12]. Indeed, Weinstein et al published in his article the results of a randomized cohort study with relatively poor results in the non-operative group [13-15]. Despite the high level of crossovers in their study, the treatment effect was favoring surgery on the SF-36 scale for bodily pain. Also Malmivaara et al showed a better recovery after surgery versus conservative treatment with a difference of improvement of 11.3 on the ODI disability scale [16]. Furthermore Turner et al published in their attempted meta-analysis a success rate (good to fair outcome) of 64% after surgical bony decompression in patients with INC [17].

Thomé et al prospectively compared the most typically used techniques: laminectomy, unilateral laminotomy and bilateral laminotomy [18]. In the series of Thomé et al, bilateral laminotomy achieved an 80% success rate. It was slightly better compared to laminectomy, which had 70% success rate [18]. Many authors claim that bony decompressive surgery might facilitate spinal fusion in the future [19,20]. Furthermore, local trauma in these surgical strategies should not be underestimated [21]. The above described operations are usually performed under general or local anaesthesia and 2 to 7 days hospitalization may be required, followed by an 8-weeks recovery period. Furthermore, the clinical outcome seems disappointing, since 35% of the patients documented bad outcome [13-17].

Minimally invasive surgery has gained popularity in recent years, resulting in the development of interspinous implants in the 1980s [22]. One of these models, the Wallis device, was made with a band around the spinous processes. Later in 2003 X-stop, in 2005 Diam, in 2006 Coflex, and afterwards various other kinds of forms were developed to stabilize or distract the interspinous distance [23-33]. These implants are all placed between spinous processes, which will lead to distraction of the interspace with consequent indirect decompression of the nerve roots. Presently, most publications refer to X-stop implants [8,23-26,29-31,34-36]. It is claimed that this indirect decompression will reduce the pressure on the nerves leading to a return to a neutral or slightly tightened position of the vertebral column. Nevertheless, this is a far smaller operation and gives perhaps less destruction to the bony elements of the vertebral column. Therefore, IPD is believed to have better short-term recovery and similar long-term (cost-) effectiveness [8,34,36,37]. Outcomes were reported to be quite favorable in selected series of poor methodological quality. The first randomized multicentre study on interspinous devices compared X-stop with non-surgical treatment [36]. After 2 years, the IPD group shows both clinically and statistically significant improved results in comparison with the conservative treated group [8,36]. However, this trial only compared IPD with conservative treatment. Good evidence on IPD versus other surgical treatment is not yet available. Verhoof et al reported in 2008 a high failure rate in IPD (X-stop), with an average slip on the radiographs of 19.6%, and a high surgical re-intervention rate (seven out of the 12) [35]. Strömqvist reported 13 re-operations in a group of 50 patients [38]. Park et al published one of the few studies with the Coflex implant [39]. However they only placed a Coflex implant after bony decompression [40]. Furthermore long term results, despite from the small retrospective series (twenty patients) of Kondrashov et al, are not yet available [34].

The golden standard in surgical treatment for lumbar spinal stenosis is bony decompression to which all new techniques should be compared. The purpose of our study is to asses whether IPD-surgery is more (cost) effective compared with surgical decompression in patients with INC due to lumbar stenosis. It is hypothesized that IPD gives particularly a favorable short term effect, necessitating a short term evaluation.

## Methods/design

An observer and patient blinded randomized (cost-)effectiveness trial in the treatment of lumbar spinal stenosis is presented. In this trial two surgical techniques are compared in a parallel group design. The primary outcome measurement is the Zurich Claudication Questionnaire. The follow-up period will last 1 year. In order to collect enough patients, a multi-center design is necessary. The study protocol was approved in all participating hospitals (see table 1: list of hospitals).

**Table 1:** list of hospitals participating in the Felix Trial

N	Hospital
1	Leiden University Medical Centre
2	Medical Centre Spaarne, Hoofddorp
3	Medical Centre Rijnland, Leiderdorp
4	Medical Centre Diaconessenhuis, Leiden
5	Medical Centre Haaglanden, The Hague
6	Medical Centre Bronovo, The Hague
7	Medical Centre Groene Hart, Gouda
8	Medical Centre Reinier de Graaf, Delft
9	Medical Centre Vlietland, Schiedam
10	Medical Centre Canisius Wilhelmina, Nijmegen
11	Medical Centre Haga, The Hague
12	Medical Centre Isala, Zwolle
13	Medical Centre Alkmaar
14	Medical Centre Tergooier, Hilversum
15	University Medical Centre Leiden

N, number of participating hospital in order of participating in this Felix Trial

Our primary question is whether IPD-surgery is more (cost-)effective compared with surgical decompression after 8 weeks in people with intermittent neurogenic claudication due to lumbar stenosis. The main advantage of IPD might be a faster recovery after surgery, but after long term follow-up it is unknown if this treatment effect will remain. Therefore, in addition, long-term follow-up (one year) will be compared with short-term follow-up.

### Patients

All patients between 40 and 85 years with at least three months of INC due to spinal canal stenosis are eligible for this study. Imaging studies (MRI) must confirm a narrowed lumbar spinal canal, nerve root canal or intervertebral foramen at one or two levels. Patients have received at least three months of conservative therapy. Lumbar discectomie is not possible during IPD surgery. Therefore, patients should be excluded when a surgical relevant herniated disc is present. Additional inclusion and exclusion criteria are listed in table 2 (inclusion and exclusion criteria).

**Table 2:** inclusion and exclusion criteria

Exclusion/Inclusion	Reason
Patient will be excluded	signed informed consent
	40 to 85 years
	has INC, as noted by leg/buttock/groin pain with or without back pain
	at least three months conservative treatment
	has a regular indication for surgical intervention INC
	has a narrowed lumbar spinal canal, nerve root canal or intervertebral foramen at one or two levels confirmed by MRI
	is physically and mentally willing and able to comply with, or has caregiver why is willing and able to comply with, the post-operative evaluations
Patient will be included	has a cauda equina syndrome
	has a herniated disc at the same level, necessitating lumbar discectomy
	has Paget's disease, severe osteoporosis or metastasis to the vertebrae
	has significant scoliosis (Cobb angle >25 degrees)
	has had previous surgery of the same lumbar level
	has degenerative spondylolisthesis > grade 1 (scale 1 to 4) at the affected level
	has significant instability of the lumbar spine
	has severe co morbid conditions
	has a fused segment at the indicated level

Patients are referred by a neurologist with MRI and conventional imaging of the lumbar spine. During the first visit to the neurosurgical outpatient clinic, the patient's history and a standard neurological examination will be documented. Conform our selection criteria, the neurosurgeon decides whether a patient is eligible for the Felix (Foraminal Enlargement Lumbar Interspinous distraXion) trial and informs the patient about both surgical techniques. The study, with both treatment options, will be explained to patients and, in case of a positive reaction, appointments are made with research nurses. Because the patient needs some time to consider participation, the first visit to the research nurse is planned after at least 2 days. After informed consent, the questionnaires, outcome measures and baseline variables are recorded.

### Ethical considerations

In concordance to the decloration of Helsinki, the study has been reviewed by an independent ethical committee and approved as being ethically constituted. The design of this study is approved by the Leiden Ethical Medical Committee. Every participating center independently needs an approval before they may include patients for this trial. Freely given informed consent will be obtained from a patient before inclusion in this study. This means that a patient has the right to know that he is being asked to take part, and that he does not have to do so unless he chooses. The patient will also be informed that there will be no financial rewards if he or she agrees to participate.

### Randomization procedure

Patients will be randomly allocated to either IPD or conventional decompression. Randomization will take place in the operating room within 4 weeks after inclusion by the research nurse. A randomized block design, stratified by hospital and research nurse, is used to ensure equal distribution of both treatments while ensuring by imposing a variable, random block size that the next treatment is not predictable for the surgeon. The randomization was prepared by the study statistician and the principle data manager at the department of Biostatistics. They were not involved in the selection and allocation of patients and prepared coded, sealed envelopes containing the treatment allocation. In the operating room, after induction of anesthesia, the surgeon will open the envelope and the allocated treatment will be performed. Patients, nursery department and research nurses are kept blinded for the allocated treatment during the follow-up period of 1 year. The operation report will be kept separately and will only be available in case of complications or reoperations.

### Interventions

After the induced general anesthesia, randomization in group (A) IPD and (B) surgical decompression will be performed. The patient is positioned in knee-elbow position or prone, dependent by the preference of the surgeon. The affected spinal level is verified fluoroscopically. The participating surgeons have experience in both techniques and performed at least five implant operations and 15 bony decompression operations.

#### A) IPD

A median lumbar incision is made over the spinous processes, the laminae of the affected level(s) are exposed subperiosteally, and the supraspinous ligament will be incised. The interspinous ligament of the affected level is removed. No decompression will be performed and the ligamentum flavum will remain intact. A Coflex™ device is placed in the created space between the spinous process with insertion of instrumentation. The wound will be closed in layers with a suction drain. The titanium Coflex™ implant that fits between the spinous processes of the lumbar spine is comprised of two components: a wing assembly and a spacer assembly. The Coflex™ is available in 5 sizes: 8 mm, 10 mm, 12 mm, 14 mm and 16 mm. The size refers to the minor diameter of the oval spacer assembly of the Coflex™. Patients will be operated with loupe magnification or microscope depending surgeon's preference. When an IPD fails, a standard laminotomy will be performed.

#### B) Surgical decompression

Similarly as in group A, a median lumbar incision will be made and the paravertebral muscles will be dissected subperiosteally and retracted bilaterally. Decompression will be applied via partial resection of the affected laminae and no complete laminectomy will be performed. The lateral recess will be opened bilaterally and medial facetectomy will be performed in order to maintain stability of the segments. The wound will be closed in layers with a suction drain. Like in the IPD group, patients will be operated with loupe magnification or microscope depending on the surgeon's preference.

The patient will be allowed to leave the bed and walk without aid on the day of surgery. If the patient regains his/her physical function, the patient will be discharged. In both studies, patients and their guided physiotherapists are stimulated to resume home activities and work as soon as possible. The latter are blinded for the allocated treatment arm as well.

### Baseline data

The baseline questionnaire assesses demographics, hobbies, sports, work status, smoking status, low back pain history, family history of INC, co-morbidity, weight and length. The patient's satisfaction at work will be registered. The patient's and the surgeon's treatment preference for IPD or decompression surgery will be assessed on a 5-point scale ranging from "strong preference for IPD" to "strong preference for decompression surgery".

### Outcome assessment

The validated outcome parameters described below will be used in this study and assessed by means of questionnaires. Follow-up examinations by the research nurse will take place at 2, 4, 8 weeks, 3, 6, 12, 24 and 60 months after randomization (see table 3: flowchart). Patients will be neurologically examined (at 8 weeks, 6, 12, 24 and 60 months) and the main questionnaires will be filled out at home with a request to complete and return them. The outpatient control by the neurosurgeon will be at 8 weeks and more often if necessary (see table 3: flowchart).

**Table 3:** Flowchart

Obtained patients' information	V1	V2	V3	V4	V5	V6^a^	V7	V8	V9	V10
In-patient		x								
Out-patient	x				x	x	x	x	x	x
Demography & diagnosis	x									
Basic physical examination	x									
Neurological examination	x				x		x	x	x	x
Provide study information	x									
Obtain informed consent	x									
X-ray		x						x		
Randomisation		x								
ZCQ	x		x	x	x	x	x	x	x	x
MRDQ	x		x		x	x	x	x	x	x
Shuttle Walking Test	x				x	x	x	x	x	x
SF-36	x			x	x	x	x	x	x	x
McGill Pain Questionnaire	x				x	x	x	x	x	x
VAS for legs and back	x		x	x	x	x	x	x	x	x
Perceived Recovery					x	x	x	x	x	x
Patient Global Impression of change					x	x	x	x	x	x
EuroQol & VAS Quality of Life	x		x	x	x	x	x	x	x	x
Patient diary					x	x	x	x	x	x
Review MRI	x									
Complications		x	x	x	x		x	x	x	x
Re-operation					x	x	x	x	x	x

A, questionnaires will be sent per mail with request to complete and return them; V1, Visit 1 - Intake; V2; Visit 2 - surgery; V3, Visit 3 - Follow-up 2 weeks; V4, Visit 4 - Follow-up 4 weeks, V5, Visit 5 - Follow-up 8 weeks; V6, Visit 6 - Follow-up 3 months; V7, Visit 7: Follow-up 6 months; V8, Visit 8 - Follow-up 12 months; V9, Visit 9 - Follow-up 24 months; V10, Visit 10 - Follow-up 60 months

#### Primary outcome measurement

The disorder-specific functional score will be the primary outcome measure and can be obtained by completing the ZCQ, also known as the Brigham Spinal Stenosis Questionnaire and Swiss Spinal Stenosis Questionnaire [41-43]. The ZCQ scale consists of 3 subscales: symptom severity, physical function and patient satisfaction. Domain scores ranges from 1 to 5, 1 to 4, and 1 to 4 respectively. Like in the study of Tuli in 2006, we chose threshold scores for each scale based on prior work [41-44]. In the symptom severity scale and in the physical function scale the minimal clinically important difference (MCID) is 0.5. A mean patient satisfaction score of less than 2.5 has been shown previously to represent a satisfied patient [42,43]. Despite from the subscale analysis we dichotomize "succes" and "failure". When the MCID threshold was achieved in at least two domains, it was described as an overall succes [44].

#### Secondary outcome measurements

##### 1) Modified Roland Disability Questionnaire for Sciatica (MRDQ)

The 23-points MRDQ is the most widely used patient-assessed measure of health for low back pain and leg pain [45-52]. This questionnaire consist of 23 questions with higher scores indicating increased disability [53]. Patrick et al compared MRDQ to patients satisfactory after from a change of 5 or more, patients feel themselves better. From a change of 12.4 all symptoms are completely gone. Others used a change of 4 or more [7]. The MRDQ will be dichotomized in "good result" (change of 4 or more) and "poor result" (change of 4 or less) [49-51].

##### 2) Shuttle walking test (SWT)

In this test a distance of ten meters has to be walked by the patients in a certain amount of time. This interval will be shortened until the patient does not finish the ten meters in the prescribed time. The SWT needs to change by 76 meters to ensure that walking distance is changed, but large changes can occur after surgery, and the SWT may thus provide a useful measure on an individual basis [54].

##### 3) SF-36

The questionnaire consists of 36 items on physical and social status of the patient subdivided in 8 domains: physical function, physical restrictions, emotional restrictions, social functioning, somatic pain, general mental health, vitality, and general health perception. The questions are scored on a scale of 0, "worst health", to 100, "ideal health" [55,56].

##### 4) McGill pain questionnaire

This score distinguishes three dimensions of pain: sensoric, affective and evaluative dimension [57,58].

##### 5) Visual Analogue Scale (VAS) score of back pain and leg pain

This parameter will measure the experienced back and leg pain intensity in the week before visiting the research nurse. Pain will be assessed on a horizontal 100 millimeters scale varying from 0 millimeter, "no pain", to 100 millimeters, "the worst pain imaginable" [59].

##### 6) Likert scale

This 7-point perceived recovery scale varies from "completely recovered" to "worse than ever". Like the patient global impression of change, the scale will be completed by the patient and research nurse. For analysis purposes this test will be dichotomized in "recovered" and "not recovered" [60].

##### 7) Hospital Anxiety Depression Scale (HADS)

This scale consists of a 7-item depression scale and a 7-anxiety scale. The score range from 0-21 with a high score being indicative for depression/anxiety.

#### Costs

To estimate utilities the EuroQol is used [61-64]. The EuroQol consists of 5 dimensions: mobility, self-care, daily activities, pain/discomfort, and anxiety/depression. Together with the remaining life expectation, they form QALY's. The QALY is a measure for the number of years someone still may expect, corrected for their quality. The EuroQol will be repeated once every two weeks during the first 8 weeks after surgery. These frequent EuroQol measurements during the first 8 weeks have been chosen in order to record the changes of quality of life. After this first period EuroQol will be recorded on regular basis during the patient's visit to the research nurse (see table 3: flowchart). The patients are also instructed to record a diary in which, for example, work activities will be enlisted. Furthermore direct medical costs will be estimated on basis of the cost centre method.

#### Complications and re-operation incidence

The research nurse and the neurosurgeon will record complications accurately. This may include infections, post-surgical haematoma, cerebrospinal fluid leakage, an increase in neurological deficit due to surgery, venous thrombosis and other side effects.

### Sample Size

**Figure 1:**
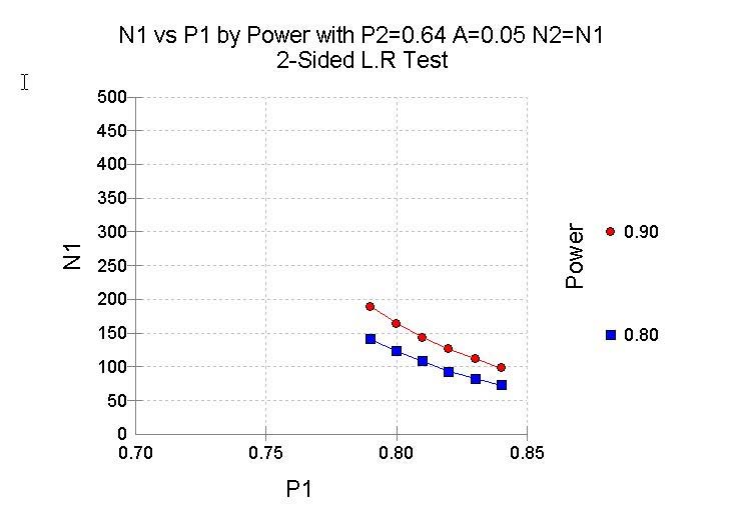
**Sample Size**. N1: number of patients needed in the IPD group, P1: the chosen succes rate of the IPD group; P2: the succes rate of the decompression group (0.64); N2: number of patients needed in the decompression group (equal to N1); A: the alfa is two sided 0.05.

The sample size calculation is based on the hypothesis that the short-term results obtained after IPD are equal to the results obtained after surgical decompression. The ZCQ at eight weeks will be used as a primary result measure both to answer the first research question and to calculate the sample size. The sample size of the trial is based on a superiority design and calculated under the alternative null-hypothesis to reach sufficient power to enable a distinction between the two arms in terms of success according to ZCQ if (according to the literature) results obtained after surgical decompression will be 64% and the results obtained after IPD will be at least 84% (20% difference in favor of IPD). A sample size of 98 patients per group ensures 90% power to confirm the null hypothesis when IPD is more than 20% superior to decompression, using a likelihood ratio test in a logistic regression framework (see figure 1: sample size). Accounting for about 10% loss to follow-up, this trial will enroll 216 patients with INC (108 patients in both groups). A sample size of 80 patients per group (including 10% loss to follow-up) will ensure a power of 80%. The feasibility of reaching 216 patients available for analysis will be checked after reaching 160 evaluable patients without deblinding or even analyzing the data as a group comparison. This constitutes a methodological valid approach since no multiple testing is involved and stopping further accrual is not based on an intermediate effect estimate. Since the power is based on a dichotomization of the underlying ZCQ scale, an alternative primary analysis of the ZCQ itself will also have sufficient power. The latter analysis will also take the repeated measurements structure into account.

### Statistical analysis

Baseline comparability will be assessed by descriptive statistics to determine whether randomization was successful. Differences in outcome between both groups, together with 95% confidence intervals, will be calculated. Besides a difference in recovery between the two groups at two specified time points (eight weeks and one year), analysis of a difference in time to recovery will be carried out as well, using a survival analysis framework (COX hazards). All data are analyzed according to the "intention-to-treat-principle". Furthermore a repeated measurements analysis of variance will be performed on the underlying continuous scales. In all analyses the first assessment of treatment effect will be the estimate of the main effect within the appropriate model, adjusted for the stratification factors and main covariates. Secondly, an interaction term evaluating a possible effect modification of the treatment effect by the major covariates (see table 4: covariates for sub analysis) is pre-specified as being part of proper statistical modeling of the primary treatment effect. In the presence of severe interaction, the treatment effect will be presented as a function of the effect modifiers. In addition, an explorative subgroup analysis is conducted to investigate whether treatment effect varies over specific subgroups of patients (table 5: subgroups). Data will be stored via the internet-based secure data management system ProMISe of the department of Medical Statistics and Bioinformatics. The analyses will be carried out using appropriate statistical software (e.g. SPSS, version 17).

**Table 4:** Covariates for subanalysis

N	Covariates for subanalysis
1	Age and age banding ( < 60 years, > 60 years or similar linked to groups size after recruitment)
2	Long medical history of back pain
3	Leg pain intensity
4	Proportion leg pain/back pain
5	Extent of stenosis during MRI examination
6	Kind of stenosis (soft or bony)
7	Sexe
8	Surface area of spinal canal

N, number of covariate (alphabetically ordered)

**Table 5:** Subgroups

Subgroups	Variables
Demographics	age < 70 years versus > 70 years
	women versus men
Anamnestic and neurological variables	short versus long history of back pain
	more leg pain versus more back pain
Radiological variables	soft versus bony stenosis
	extent of stenosis during MRI examination

Subgroups bases on the following variables.

## Discussion

In this article a design of a RCT is presented which evaluates the (cost-) effectiveness of IPD versus decompression surgery in the treatment of intermittent neurogenic claudication. This is the first randomized prospective trial comparing these two surgical techniques. Like the Sciatica-MED trial, the research nurse and the patient are blinded for the allocated treatment [7]. The objective of this trial is to determine whether the IPD is more (cost-) effective after eight weeks compared to the conventional decompression surgery.

## Abbreviations

CRF: case record form; INC: intermittent neurogenic claudication; IPD: interspinous process device; MCID: minimal clinically important difference; MRDQ: modified Roland disability questionnaire; MRI: magnetic resonance imaging; QALY: Quality adjusted live years; RCT: Randomized Controlled Trial; SF-36: short form-36; SWT: Shuttle walking test; VAS: visual analogue scale; ZCQ: Zurich claudication questionnaire.

## Competing interests

WPE received a grant from Paradigm Spine and InSpine to create and carry out the Trial

## Authors' contributions

WAM is the coordinator and principal investigator of the trial. WCP and MPA designed the study protocol and are the supervisors of WAM. RB is the responsible biostatistician and also responsible for the implementation of the trial data management using the ProMISe software. BWK is the epidemiological supervisor. He will be analyzing the data together with WAM. All authors participated in the trial design and coordination. All authors read and approved the final manuscript.

## Pre-publication history

The pre-publication history for this paper can be accessed here:

http://www.biomedcentral.com/1471-2474/11/100/prepub
